# Oligonucleotide Solid Nucleolipid Nanoparticles against Antibiotic Resistance of ESBL-Producing Bacteria

**DOI:** 10.3390/pharmaceutics14020299

**Published:** 2022-01-27

**Authors:** Phuoc Vinh Nguyen, Clémentine Aubry, Narimane Boudaoud, Alexandra Gaubert, Marie-Hélène Langlois, Mathieu Marchivie, Karen Gaudin, Corinne Arpin, Philippe Barthélémy, Tina Kauss

**Affiliations:** 1 ARNA, Inserm U1212, CNRS 5320, University of Bordeaux, 146 rue Léo Saignat, CEDEX, 33076 Bordeaux, France; phuocvinhnguyen.pharm@gmail.com (P.V.N.); clementine.aubry@u-bordeaux.fr (C.A.); narimane.boudaoud@u-bordeaux.fr (N.B.); alexandra.gaubert@u-bordeaux.fr (A.G.); marie-helene.langlois@u-bordeaux.fr (M.-H.L.); karen.gaudin@u-bordeaux.fr (K.G.); philippe.barthelemy@inserm.fr (P.B.); 2 UMR 5026, University of Bordeaux, CNRS, Bordeaux-INP, ICMCB, 87 Avenue du Dr Albert Schweitzer, CEDEX, 33608 Pessac, France; mathieu.marchivie@u-bordeaux.fr; 3 MFP, CNRS 5234, University of Bordeaux, 146 rue Léo Saignat, CEDEX, 33076 Bordeaux, France; corinne.arpin@u-bordeaux.fr

**Keywords:** oligonucleotides, antibiotic resistance, solid nanoparticles, nanocapsules, ESBL-producing *E. coli*, nucleolipid, nucleic acids, CTX-M15 ß-lactamase

## Abstract

Antibiotic resistance has become a major issue in the global healthcare system, notably in the case of Gram-negative bacteria. Recent advances in technology with oligonucleotides have an enormous potential for tackling this problem, providing their efficient intrabacterial delivery. The current work aimed to apply this strategy by using a novel nanoformulation consisting of DOTAU, a nucleolipid carrier, in an attempt to simultaneously deliver antibiotic and anti-resistance oligonucleotides. Ceftriaxone, a third-generation cephalosporin, was formulated with DOTAU to form an ion pair, and was then nanoprecipitated. The obtained solid nanocapsules were characterized using FT-IR, XRD, HPLC, TEM and DLS techniques and further functionalized by the anti-resistance ONα sequence. To obtain an optimal anti-resistance activity and encapsulation yield, both the formulation protocol and the concentration of ONα were optimized. As a result, monodispersed negatively charged nanoparticles of CFX–DOTAU-ONα with a molar ratio of 10:24:1 were obtained. The minimum inhibitory concentration of these nanoparticles on the resistant *Escherichia coli* strain was significantly reduced (by 75%) in comparison with that of non-vectorized ONα. All aforementioned results reveal that our nanoformulation can be considered as an efficient and relevant strategy for oligonucleotide intrabacterial delivery in the fight against antibiotic resistance.

## 1. Introduction

Multidrug-resistant bacteria (MDR) constitute a major public health concern [1]. This situation is particularly critical among extended-spectrum ß-lactamase (ESBL) producing *Enterobacteriaceae* [1,2,3], classified by the World Health Organization as one of three pathogens of critical priority in terms of needing new therapeutic strategies [4]. ESBL-producing *Escherichia coli* (*E. coli*) were hence chosen as a model for this study. These resistant enterobacteria inactivate most ß-lactam antibiotics, the predominant antibiotic class used to treat bacterial infections in humans. ESBL genes (*bla* genes) are mainly disseminated among bacteria through conjugative plasmids, which also carry other resistance genes, leading to a “multi” and even “pan-resistance”, defined as resistance to all known families of antibiotics and consequently to treatment failure [3]. Since the 2000s, ESBLs called CTX-M have gained prominence and are considered pandemic enzymes. The name “CTX-M” refers to their potent β-lactam hydrolytic activity against cefotaxime (a reference third-generation cephalosporin, 3GC). In France, among different clinical *E. coli* isolates, group 1 CTX-M enzymes (CTX-M-15 (37.1%) and CTX-M-1 (24.2%)) are the most prevalent [3,5]. However, these CTX-M β-lactamases can also deactivate ceftriaxone (CFX), an extended-spectrum 3GC approved for once- or twice-daily treatment of patients with Gram-positive or Gram-negative infections [6], which was used in this study as a model 3GC antibiotic. CFX was initially used for severe or multi-drug resistant infections (e.g., meningitis, lower respiratory tract infections, community and hospital-acquired pneumonia, acute exacerbations of chronic bronchitis, intra-abdominal infections, urinary tract infections including pyelonephritis, bacterial endocarditis, and infections of bones and joints) [7]. Since then, CFX has also undergone resistance development, including in ESBL-producing *E. coli*, as recently reviewed [8], in which the production of ESBL was identified as a major resistance mechanism [9].

In this context, the appropriate use and dose of antibiotics [10,11], antibiotics in combination to broaden the spectrum and generate synergistic effects [12,13], and the development of new or chemically modified antibiotics have received increasing interest in the scientific community [11,13]. In addition, different therapeutic strategies have been developed, such as the use of phages, predatory bacteria, antimicrobial peptides, gene-editing enzymes and metals [2,14]. However, discovering and testing new antibiotics is a costly and time-consuming process, which explains why many major pharmaceutical companies have already given in [15,16,17].

Another way to tackle antibiotic resistance is to improve antibiotic delivery systems in order to reduce the amount and frequency of antibiotic doses or implement targeting. Among current drug delivery systems, nano-carriers have been shown to be highly potent [8,18,19,20,21,22]. Antibiotic nanoparticles (NP) reportedly [23,24] enhanced antibiotic effects via the formation of a local reservoir close to the bacterial cell wall [25]. For instance, the antibacterial activity of vancomycin was remarkably increased when it was encapsulated in NP by forming ion pairs with linoleic acid [23]. Therefore, the formulation of CFX in ion pairs NP appears as a potential solution to enhance its antibacterial activity.

To form ion pairs with CFX using the nanoprecipitation method, nucleolipids can be employed due to their potent properties in terms of protecting the formulated drug and enhancing its intracellular delivery. Indeed, nucleolipids were shown to possess intrinsic molecular recognition and favorable cell-penetrating abilities [26]. DOTAU nucleolipid has already been used to successfully formulate solid lipid NP [27,28] and was selected for ion pairing and as a nanocarrier in this study.

In addition to an optimized antibiotic delivery, recent trends have shown the potential of oligonucleotides as antibacterial agents, either to target viable genes and, hence, kill the targeted bacteria, or to target resistance genes and decrease bacterial resistance. Among a variety of chemically modified oligonucleotide backbones, peptide nucleic acids (PNA) chemistry has mainly been used, but applications of phosphorothioate oligonucleotides (PTO), locked nucleic acids (LNA) and phosphorodiamidate morpholino-oligomers (PMO) have been described [29,30,31,32]. The application of PTO antisense oligonucleotides (ASO) to bacteria demonstrated their effective activity in reducing minimum inhibitory concentrations (MICs) in *Mycobacterium tuberculosis* [33] and was shown to be efficient in the inhibition of gene expression in *Escherichia coli* [34]. PMO and PNA oligonucleotides were shown to be efficient in partially restoring cefotaxime activity in ESBL *E. coli* [35].

Our recent work [36] demonstrated the interest of the use of oligonucleotides to decrease the expression of periplasmic ß-lactamases CTX-M-15, at the origin of ESBL-E resistances. Literature-based [35] and lab-designed sequences, synthetized in PTO structures and conjugated or not to lipids, were considered for the CFX MIC decreasing of laboratory and clinical strains of ESBL *E. coli* [36].

However, the main challenge for the use of oligonucleotides in bacteria is to ensure an efficient cell penetration. Several approaches have been described in the literature including DNA-built tetrahedra [37] or Ag-DNA nanodevices [38], oligonucleotide chemical modifications including cell-penetrating peptide conjugation [29,30,35,39,40] or nucleolipid conjugates [36], and nanodelivery using liposomes [41,42,43], lipoplexes [44], conjugated polymers [44] or cationic polymers [44,45,46,47,48,49].

The aim of the current study is to evaluate and optimize the potential of a novel nanoformulation in decreasing the MIC of ESBL-producing *E. coli*. The nanoformulation design simultaneously combines (i) the PTO oligonucleotide against *bla*_CTX-M-15_, (ii) ceftriaxone as a model 3GC antibiotic, associated with (iii) membrane penetration enhancing nucleolipid DOTAU as a carrier.

## 2. Materials and Methods

### 2.1. Material

Ceftriaxone heptahemihydrate di-sodium salt of pharmaceutical grade was purchased from Discovery Fine Chemical (Wimborne, UK).

DOTAU chloride was synthetized in our laboratory [48] by a Technology Transfer Unit SynVec (Bordeaux, France). diC16dT nucleolipid (sodium salt) was synthetized in-house [49].

Methanol and Acetonitrile (HPLC grade) were purchased from VWR (Fontenay-sous-Bois, France).

Demineralized water was prepared at the laboratory by ion exchange (Pure Lab Option ELGA) followed by distillation (Water Still Distinction D4000).

Bacterial *E. coli* strains used were sensitive and transconjugant K12 *E. coli* with resistance gene plasmids coming from *Ec3536* (with *bla*_CTX-M-15_ plasmid [5], such as that described in our previous work [36]).

Microbiologically consumable Mueller–Hinton bacteria culture medium, adjusted in calcium and magnesium ions (MH-CA), was purchased from Bio-Rad, Paris, France.

### 2.2. Synthesis and Purification of Oligonucleotides

The proof of concept was performed using a previously described sequence [36], which was complementary to *bla*_CTX-M-15_: 5′-GCG CAG TGA TTT TTT AAC CAT GGG A-3′. The sequence was synthetized using PTO backbone and named oligonucleotide α (ONα).

Briefly, an automated synthesis was performed using H8 DNA Synthesizer (K&A Laborgeraete, Schaafheim, Germany) at the µmolar scale on 1000 Å primer support (loading: 30–100 µmol·g^−1^, Link Technologies, Synbase Control Pore Glass) with conventional β-cyanoethyl phosphoramidite chemistry. Phosphorothioate linkage was introduced during the synthesis cycle using Sulfurizing Reagent II (3-((N,N-dimethylaminomethylidene)amino)-3H-1,2,4-dithiazole-5-thione from Glen Research).

The purification used previously described HPLC analysis and HPLC preparation methods [36] followed by dialysis in Spectra-Por 6 dialysis membranes (MWCO 1kD) in distilled water (3 times 1 L for 1 h). The identity of the sequence was confirmed using previously described and reported MS [36].

The concentration of the oligonucleotide samples was measured by microvolume spectrophotometer (mySPEC, VWR^®^) at 260 nm using automatic oligonucleotide detection mode.

### 2.3. Preparations of Ion Pairs, CFX–DOTAU and ONα Nanoparticles (NP)

Ion pairs were obtained by the vortex mixing of CFX extemporaneously prepared aqueous solution and DOTAU sonicated aqueous dispersion at variable concentrations and volumes. White precipitated ion pairs were then centrifuged for 2 min at 5031× *g* (Minispin plus, Eppendorf) and washed twice with 1 mL of demineralized water. The pellet was dried in (Digital Heatblock VWR) at 30 °C under air flow. The dried film was re-dissolved in 300 µL of methanol using vortexing.

#### Nanoprecipitation Technique

CFX–DOTAU NP were obtained by means of the nanoprecipitation method. Methanolic ion pair solution was added dropwise, using a syringe (Terumo^®^ Syringe 1 mL) and a needle (Fine Ject^®^ 25 G × 5/8″ 0.5 × 16 mm), into a glass tube containing 10 mL of demineralized water under vortex agitation with a constant speed of 1 drop every 2 s. Methanol and demineralized water were evaporated using a Heidolph Rotary Evaporator (Laborota 4001) at 40 °C to obtain a final volume of NP suspension of 1 mL.

ONα NP were obtained through the incubation of CFX–DOTAU NP in ONα solutions (100, 200, 300, 400, 600, 800 µM) for 30 min (unless stated otherwise for protocol optimization) at room temperature. The cited concentration of ONα refers to the concentration of the stock solution during the incubation and not to the concentration in the testing conditions. They were named accordingly (e.g., ONα_600_ NP for NP prepared with 600 µM ONα solution; ONα NP referring generically to Onα-functionalized NP).

### 2.4. Characterization of Ion Pairs, CFX–DOTAU and ONα NP

#### 2.4.1. IR Analysis of Ion Pair and Raw Materials

A Perkin Elmer Fourier Transform-Infrared (FT-IR) apparatus was used in Attenuated Total Reflection (ATR) mode. Dried and crushed ion pairs were placed on a diamond after the acquisition of background signal. All spectra were an accumulation of 8 spectra with a resolution of 8 cm^−1^. The Spectrum software was used to treat results.

#### 2.4.2. X-ray Diffraction Analysis

The structure of the complex was studied using powder X-Ray Diffraction (XRD) and patterns were collected on a PANalitycal X’pert PRO MPD diffractometer with Bragg–Brentano θ-θ geometry equipped with a secondary graphite monochromator and an X’Celerator multi-strip detector. Each measurement was made within an angular range of 2θ = 4–38° and lasted for 100 min. The Cu-Kα radiation was generated at 45 KV and 40 mA (λ = 0.15418 nm).

The samples were put on sample holders made of aluminum alloy and flattened with a piece of glass. CFX and DOTAU raw material were used as controls. 

#### 2.4.3. Analysis of CFX and DOTAU Content for Yield and Molar Ratio Determination

The high-performance liquid chromatography (HPLC) method for CFX was adapted from previously developed and validated methods for CFX [50] and DOTAU [51], respectively. Briefly, a UHPLC UltiMate 3000 from Dionex-Thermo Scientific (Darmstadt, Germany), composed of a pump with a quaternary valve, a thermostated auto-sampler, and a thermostated column compartment was used. Two different conditions were used for the analysis of CFX and DOTAU. The injection volumes were set at 5 and 1 µL for CFX and DOTAU, respectively. A Diode Array Detector (DAD 3000) was used at 240 and 255 nm for CFX and DOTAU, respectively. The RP-HPLC columns were a J’sphere^®^ ODS-H80 (4.6 × 150 mm id, 4 µm) + a guard column ODS-H80 (10 × 4 mm id, 4 µm) (Interchim, Montluçon, France) and an Acquity^®^ UPLC BEH C18 (50 × 2.1 mm, 1.7 μm) (Waters, Milford, MA, USA) for CFX and DOTAU, respectively. The mobile phase for the CFX method was a mixture of 60% aqueous phase prepared by dissolving 25 mM of C16-TMA Br in phosphate buffer of 25 mM at pH 7.5 with 60% acetonitrile, at 1 mL·min^−1^. The mobile phase for the DOTAU method was composed of 100% methanol containing 20 mM of ammonium acetate at 0.5 mL·min^−1^. 

The ion pairs’ formulation yield and CFX–DOTAU NP yield were calculated as follows:

(1) $$\mathrm{Ion}\;\mathrm{pair}\;\mathrm{Yield}=\frac{\mathrm{mass}\;\mathrm{of}\;\mathrm{film}}{\left(\mathrm{mass}\;\mathrm{of}\;\mathrm{CFX}+\mathrm{mass}\;\mathrm{of}\;\mathrm{DOTAU}\right)}\times 100\%$$

(2) $$\mathrm{NP}\;\mathrm{Yield}=\frac{\mathrm{mass}\;\mathrm{of}\;\mathrm{NP}\;\left(\mathrm{calculated}\;\mathrm{from}\;\mathrm{HPLC}\right)}{\left(\mathrm{mass}\;\mathrm{of}\;\mathrm{CFX}+\mathrm{mass}\;\mathrm{of}\;\mathrm{DOTAU}\right)}\times 100\%$$

Molecular ratios of CFX–DOTAU ion pair or NP were directly calculated from the HPLC-defined molar concentrations of 3 samples.

#### 2.4.4. Characterization of NP Size, Polydispersity Index (PDI) and Zeta Potential

The size, polydispersity index (PDI) and Zeta potential of NP were measured using the Zetasizer Nano ZS90 (Malvern Instruments Ltd., Worcestershire, UK). Size was measured in a specific cell ZEN 0040 and expressed as Z-average mean size, and Zeta Potential in a DTS 1070 cell (Malvern Panalytical, Palaiseau, France). Measurement conditions were performed in demineralized water, at the temperature of 25 °C achieved after the equilibration time of 120 s. Each test was triplicated.

Unless otherwise stated, samples were diluted 1/100 in demineralized water to reach the desired concentration for analysis. 

#### 2.4.5. TEM Analysis

Images were acquired with a Hitachi H 7650 electron microscope. Quantities of 6 µL of NP suspension were deposited on carbon film grid for 2 min 30 prior to drying at room temperature. Contrast was applied using Uranyless for 2 min before drying. For compositional mapping and energy-dispersive-X-ray spectroscopy (EDS), a ThermoFisher Talos F200S G2, operated at 200 kV, was used combined with a STEM (scanning/transmission electron microscopy) unit and a STEM-HAADF (high angle annular dark field) detector. X-EDS spectra were accumulated for 2 min and compositional mapping was performed for 15 min on uncontrasted samples. VELOX software was required for data acquisition and processing.

#### 2.4.6. Stability of ONα NP upon Dilution

Concentrated samples of CFX–DOTAU and ONα NP equivalent to 4096 mg·L^−1^ CFX were diluted in demineralized water to 1/100, 1/200, 1/500 and 1/1000. DLS and zeta potential were measured as described above.

### 2.5. MIC Determination of NP in ESBL-Producing E. coli

Determination of CFX MIC of ONα NP was performed on K12 transconjugant strain of *E. coli* with a conjugative plasmid carrying the *bla*_CTX-M-15_ gene from the clinical strain Ec3536 [36].

Free CFX, ion-pair NP and ONα were used as control conditions.

MICs were determined by the broth micro-dilution method in accordance with the standard conditions [52].

The bacterial inoculum was prepared in 0.85% NaCl from 24 h colonies on plates at an equivalent of 0.5 of the McFarland measurement (Densimat, BioMerieux). Bacterial suspension was diluted in MH-CA 2X broth (MH, BioRad) to obtain 5 × 10^4^ cfu at the final volume of 100 µL. CFX of NP, labelled in terms of their equivalent CFX content values (serially diluted 2-fold), were added in order to obtain a final volume per well of 96-well microplates of 50 µL and completed with 50 µL of 2× ONα nanoparticle suspension. Two dosage ranges, starting at 2-fold serial dilutions from 2048 mg·L^−1^ and 1536 mg·L^−1^ of CFX respectively, were interposed to decrease the standard deviations observed. Microplates were incubated at 35 ± 2 °C for 24 h. The MICs were recorded as the lowest concentration that inhibited visible growth observed in the wells measured using a turbidimeter (Apollo LB 911 (Berthold)) at 620 nm (optic density > 0.1). Independent MIC experiments were repeated at least in triplicate.

### 2.6. Statistical Analysis

Statistical analysis was performed using the Student t-test. Each experiment was performed at least in triplicate. Data were expressed as the mean ± standard deviation (SD). Unless otherwise stated, *p* < 0.05 (*) or *p* < 0.01 (**) were considered as significant.

## 3. Results and Discussion

### 3.1. CFX DOTAU Ion Pair Formation

**Figure 1:**
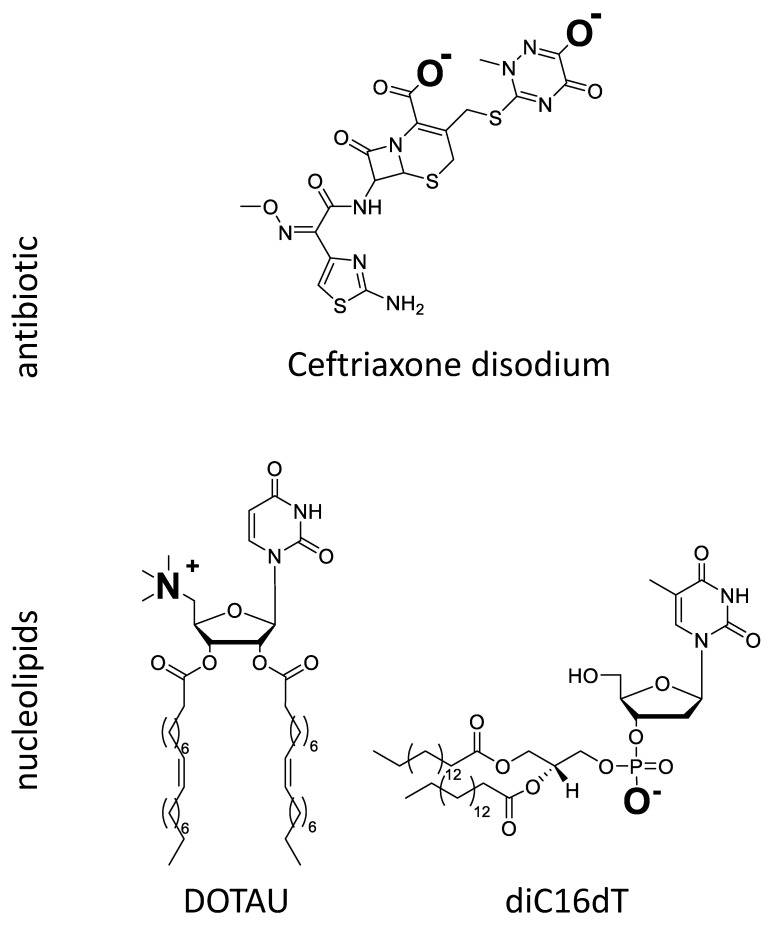
Chemical structure of CFX and nucleolipids (DOTAU and diC16dT) ionized in aqueous medium at neutral pH.

The first objective was to form an ion pair of CFX, which would reportedly [53,54] enhance its permeability. Nucleolipid DOTAU was selected as a candidate due to its physico-chemical properties. The chemical structures are depicted in Figure 1. 

DOTAU is a modified lipid nucleoside [55], which, at physiological pH, is positively charged. In aqueous solutions at room temperature, this nucleolipid forms liposome-like structures. Given the high structural variability of this amphiphilic compound, all interactions (H-bonds, π-stacking, electrostatic, hydrophobic interactions) may contribute to the stabilization of the self-assembled aggregates [56,57,58]. DOTAU has already been used to successfully formulate solid lipid NP [27,28,59], where it promoted cell membrane penetration. In this work, it was chosen as a counter ion for CFX to formulate an ion pair with CFX.

CFX is a crystal hemi-heptahydrate di-sodium salt with three pKas of 2.37 (COOH), 3.03 (aminothiazole) and 4.21 (hydroxytriazinone), respectively [60], and is consequently negatively charged at physiological pH. Its low permeability (i.e., log P −1.7 and oral bioavailability <1% [61]) and its high hydrosolubility (freely soluble in water [62]) classify CFX among the molecules of class 3 of the Biopharmaceutical Classification System (BCS). In order to improve the absorption of CFX, ion pairs between CFX and bile salts have been reported to improve the lipophilicity [53,63] and bioavailability using non injectable administration routes [64,65,66] while preserving its antibiotic activity.

The idea of the formulation approach was to use a carrier forming an ion pair with CFX by charge interaction. The ion pairing between positively charged DOTAU and negatively charged CFX was anticipated from the opposed charges (Figure 1). When CFX aqueous solution and DOTAU aqueous dispersion were brought into contact, spontaneous precipitate was formed. A change in physicochemical properties, namely of the aqueous solubility of ion pair formed with DOTAU, was observed, leading to its precipitation. A control experiment with negatively charged nucleolipid, diC16dT (Figure 1), where no precipitate was observed, confirmed the necessity of the interaction of opposite charges. 

The formulation’s initial ratio between CFX and DOTAU was explored to enhance the ion pair yield (Table 1).

The highest ion pair yield (i.e., 80% of initial mass recovered in the film) was prepared with the molar initial ratio of 1:1 for CFX and DOTAU and was, therefore, kept for further experimentation. Laboratory scale-up was performed in view of biological tests, comparing yields of three different quantities of ion pairs formed using a 1:1 molar CFX—DOTAU ratio (8–12 mg, 17–21 mg and 23–25 mg). The yields obtained were of comparable range (80 ± 4%, 74 ± 5% and 72 ± 3%, respectively), even if a limited decrease was observed when the batch scale increased.

The characterization of the CFX–DOTAU ion pair was first performed using FT-IR and XRD analyses.

The IR spectra of DOTAU, CFX and the ion pair (depicted in Figure S1) showed that several common bands from CFX and DOTAU were found in the ion pair, attesting the presence of both components in the formulation. Interaction induced limited band shifts (not higher than 3 cm^−1^, cf. Table 2 for details) and minimal vibration modifications, indicating the preservation of the main chemical groups observed.

**Figure 2:**
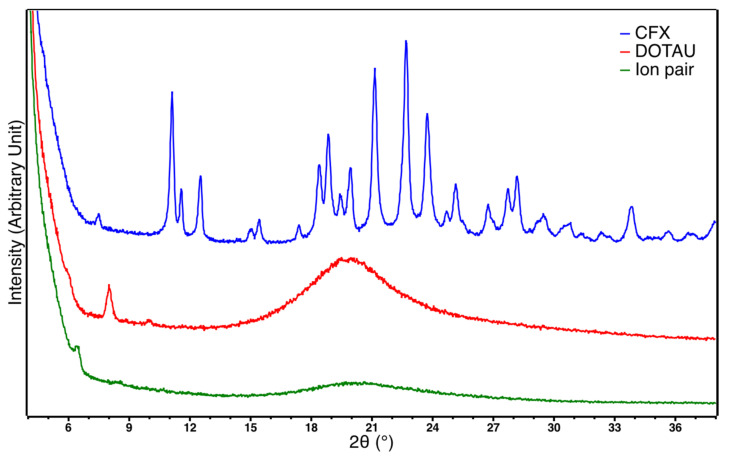
X-ray diffractogram of CFX (blue), DOTAU (red) and ion pair (green).

Powder XRD further confirmed the interaction of CFX and DOTAU as the crystalline structure of CFX became amorphous when the ion pair was formed (Figure 2). The resulting XRD diagram was indeed very similar to that obtained for pure DOTAU, showing that the ion pair adopted an amorphous structural arrangement similar to that of the DOTAU. Nevertheless, it is worth noting that a sharp peak was observed at a low angle as well for DOTAU compared to that of the ion-pair but at a slightly different position (2θ = 8.0° for DOTAU and 6.4 for the CFX–DOTAU ion pair). This peak was likely corresponding to a structural feature linked to the long aliphatic chains of DOTAU. It might correspond to a dimerization of the DOTAU leading to a dimer of approximately 44 Å length, as observed for other nucleolipids [67]. The same feature was observed on the ion-pair diagram but at a lower angle corresponding to a longer dimer of approximatively 55 Å length. This 11 Å increase was in perfect agreement with the insertion of CFX in between the polar heads of DOTAU molecules, leading to a longer dimer.

The phenomenon of amorphization during the formation of ion pairs, such as that observed in our results, is common in the literature, and has previously been described for CFX in the presence of cationic biliary salt derivatives [53,63].

### 3.2. Preparation of CFX–DOTAU NP and ONα NP

NP were prepared by dissolving the ion pair film followed by nanoprecipitation.

Among several solvents tested (i.e., methanol, ethanol, Miglyol 812 + lecithin, Miglyol 840 + lecithin, and polyethylene glycol 400), only methanol showed sufficient solvent properties for the CFX–DOTAU film (commonly dissolving 12 mg of film in 300 µL of methanol or equivalent proportion) and was kept for further formulation.

The optimal volumes of methanol and water used for nanoprecipitation were investigated (Table 3).

The volume of 10 mL of water in which the methanolic solution was nanoprecipitated allowed better encapsulation yields compared to 5 mL, and 300 µL of methanol allowed better PDI compared to 500 µL.

The CFX and DOTAU were analyzed at each step using the HPLC method, which allowed the determination of the NP encapsulation yields and the molar ratio of 1 CFX for 2.4 DOTAU in the final composition of nanoprecipitated ion pairs. The conditions of washing ion pair pellets were optimized to discard the excess CFX but preserve the ion pair formed (low quantity of DOTAU in supernatant).

**Figure 3:**
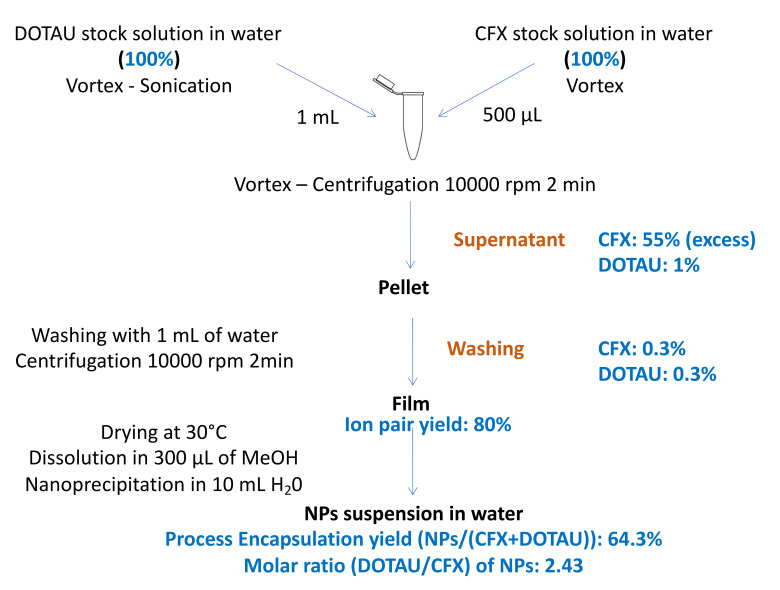
Optimized formulation protocol and HPLC analysis results (in blue).

The HPLC results and the optimized protocol are given in Figure 3.

**Figure 4:**
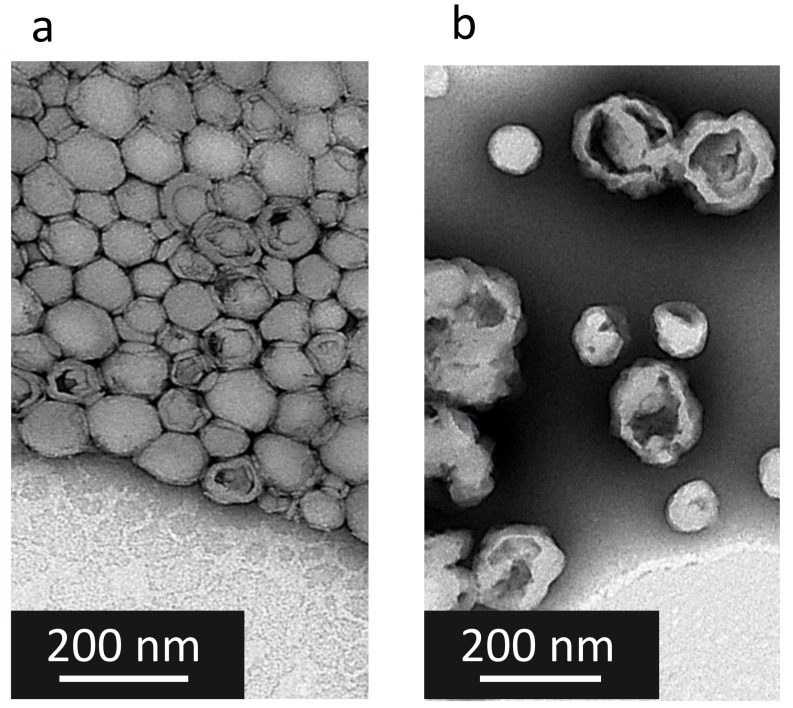
TEM images of (**a**) CFX–DOTAU NP and (**b**) Onα_600_ NP.

After the nanoprecipitation, solid monodisperse CFX–DOTAU NP of 158 nm ± 8 nm and positive zeta potential (36 mV ± 3 mV) were obtained (cf. Table 4 for details). The positive zeta potential indicated that DOTAU molecules were present at the external surfaces of the nanoparticles, providing the positive charge. The TEM images (Figure 4a) illustrated the spherical and hollow objects of nanometer range, i.e., solid nanocapsules.

The functionalization of CFX–DOTAU NP with ONα to formulate ONα NP was anticipated as the interaction of Onα-negative charges on the CFX–DOTAU-positive surface. It induced the following changes in the NP parameters: (i) an inversion of NP zeta potential from positive to negative and (ii) an increase in NP size. These changes were used to monitor the formation of ONα NP.

Monodisperse nanoparticles of mean size of 187 nm ± 21 nm and −46 mV ± 11 mV, of mean zeta potential, were formed when incubated with 600 µM ONα (cf. Table 4 for details). The TEM was performed on ONα NP, but, as expected, could not visualize ONα at the NP surface (Figure 4b). STEM mapping demonstrated that ONα NP indeed contained ONα via the presence of phosphorus (Figure S2).

**Figure 5:**
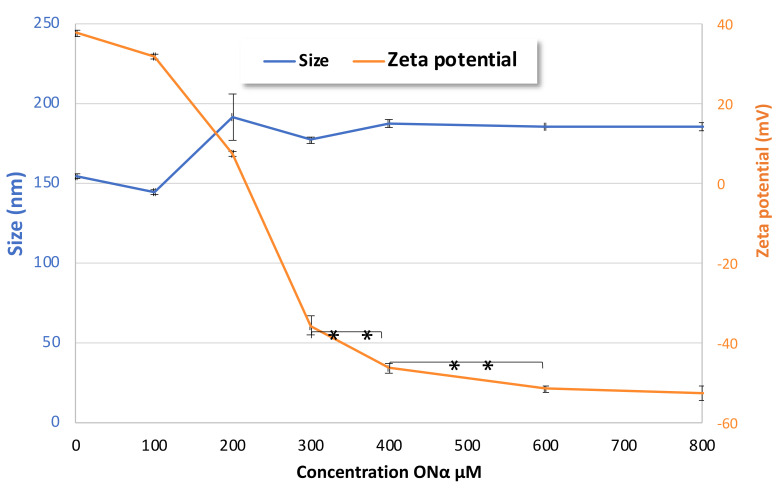
Evolution of the effects of ONα NP size and zeta potential on ONα concentration during formulation (** *p* < 0.01, Student *t*-test).

The functionalization of NPs with ONα sequences was shown to be Onα-concentration-dependent, as shown in Figure 5. The positive zeta potential of CFX–DOTAU NP (without ONα) decreased to an almost neutral level when incubated with 200 µM Onα, and became negative for higher ONα concentrations. The *plateau* of zeta potential, and hence of ONα adsorption on the ONα NP surface, was reached at 600 µM ONα.

When molar ratios were recalculated for the incubation condition of 600 µM ONα and 4096 mg·L^−1^ CFX, such as those used as a double concentrated (2×) stock formulation for bacteriological tests, the ratio of CFX:ONα was of 10:1 in the final formulation, bringing the total molar ratio of CFX:DOTAU:ONα to 10:24:1 and the inherent charge ratio to 20(−):24(+):25(−). The ion pair ratio between positively charged nitrogen of DOTAU and negative charges of CFX (N/CFX) was hence of 1.2, and gave solid positively charged capsules. The charge ratio of positively charged nitrogen from DOTAU and negatively charged nitrogen of the phosphorothioate group from ONα (N/P) (the amine group of CFX not being ionized at physiological pH) was 0.96.

**Figure 6:**
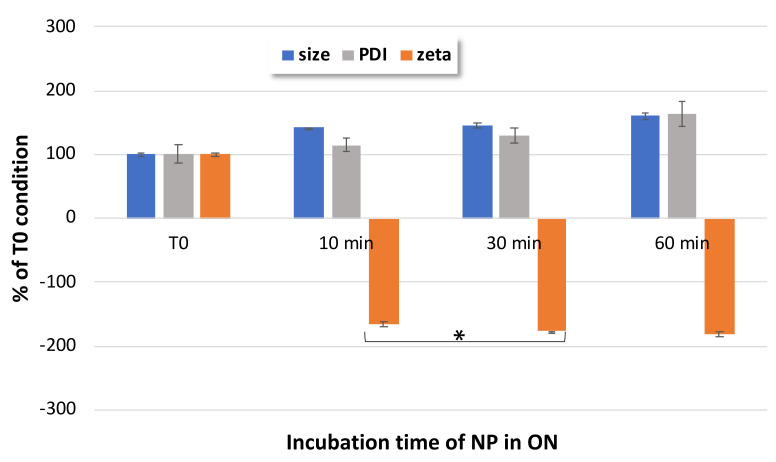
Impact of incubation time on ONα_600_ NP size, PDI and zeta potential, as compared to non-incubated CFX–DOTAU NP (at T0 time); * Student *t*-test was considered significant for *p* < 0.05.

To further optimize the formulation protocol, the impact of the incubation time in ONα solution was investigated in relation to NP characteristics, i.e., the NP size, PDI and zeta potential. Figure 6 shows that the increased size and the decreased zeta potential could be observed early, from 10 min onwards. The evolution of the NP size and PDI were not significant between 10 min and 30 or 60 min. However, the zeta potential significantly (*p* < 0.05, Student *t*-test) decreased until 30 min, indicating that at 30 min of incubation, a complete ONα adsorption was obtained. All these results led us to the conclusion that an incubation time of 30 min was needed for an optimal functionalization of our NPs with ONα.

**Figure 7:**
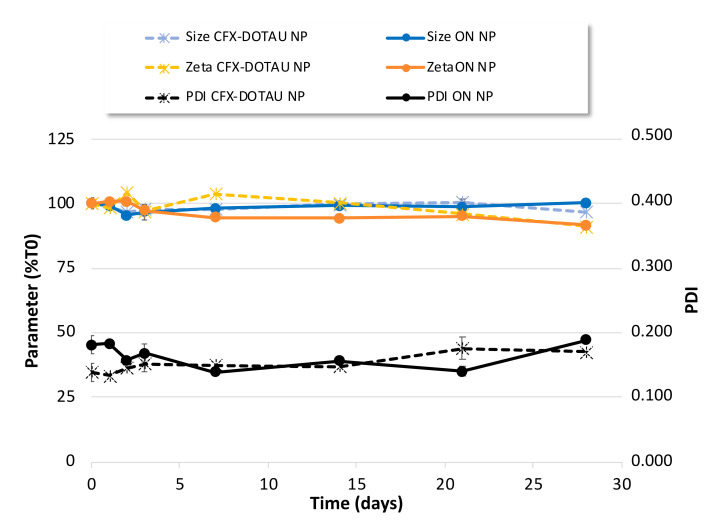
Colloidal stability of CFX–DOTAU and ONα_600_ NP characteristics (mean +/− SD): (left axis, expressed as % T0) size, zeta potential and (right axis) PDI.

The colloidal stability of NP was further investigated over 1 month at 4 °C. As shown in Figure 7, no major evolution of zeta potential or NP size was observed and the PDI values remained under 0.200 for this one-month period.

In view of further investigations requiring different concentrations of the nanoformulation, the stability of ONα NP upon dilution was questioned. Serial dilutions of CFX–DOTAU NP and ONα NP were evaluated for their size, PDI and zeta potential. As summarized in Table S1, the size and PDI of ONα_600_ NP did not significantly change (*p* > 0.05) up until 1/1000 dilution, and the zeta potential did not significantly change up until 1/500 dilution.

Altogether, these results revealed a good colloidal stability of our final NPs over time and upon dilution.

### 3.3. Evaluation of Antibacterial Effect on ESBL-Producing E. coli

**Figure 8:**
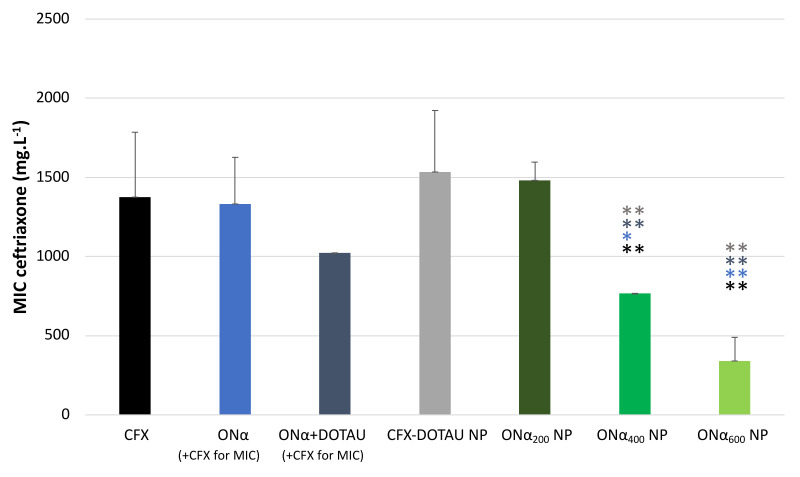
CFX MIC of ONα NP on ESBL-producing *E. coli* compared to CFX–DOTAU NP and control conditions (* *p* < 0.05 and ** *p* < 0.01 Student t-test; NB: the concentration cited in brackets is the ONα incubation concentration during NP preparation).

The MIC of ONα NP was tested on the transconjugant *E. coli* K12 and compared to the one of control conditions and CFX–DOTAU NP. As shown in Figure 8, the MIC values of CFX, of ONα in the presence of CFX, and of CFX–DOTAU NP were not significantly different (*p* > 0.43). However, a remarkably significant decrease of 75% for CFX MIC was demonstrated for the optimized formulation (i.e., incubation with 600 µM ONα). This indicated the capacity of the ONα NP formulation to efficiently vectorize the ONα and decrease the MIC and, hence, the resistance of ESLB-producing *E. coli* to the CFX antibiotic. The ONα alone (i.e., non-encapsulated), co-incubated with CFX, did not significantly modify the MIC, nor did the combination ONα with DOTAU. 

The decrease in the MIC was dependent on the ONα incubation concentration. The nanoformulation of ONα NP induced a significant decrease (*p* < 0.05 or 0.01, cf. Figure 8) in the MIC starting from the 400 µM ONα incubation concentration (ONα_400_ NP formulation), compared to the CFX and ONα control conditions and to that of CFX–DOTAU NP. The MIC of the ONα_400_ NP formulation was 768 mg·L^−1^, at which the ONα concentration, recalculated from the molar ratio, was 112 µM. The optimal results were obtained with ONα_600_ NP, giving the CFX MIC of 341 mg·L^−1^, at which the recalculated ONα concentration was 50 µM. These results were significantly lower (with *p* < 0.01) compared to the ONα_400_ NP formulation, which was in line with our mechanism hypothesis [36] that the higher number of ONα copies in bacterial cells more efficiently knocked down ß-lactamase expression, resulting in lower resistance of ESBL-producing *E. coli*.

Expectedly, ONα_600_ NP did not have any significant impact on the CFX MIC of the non-resistant, parental K12 strain (0.016 ± 0.07 mg·L^−1^), compared to CFX NP.

Our previous work [36], using fluorescent microscopy, showed that the lack of *E. coli* penetration of non-formulated PTO ONα appeared to be the reason for the non-modified MIC, whereas chemically modified nucleolipid-conjugated Onα, upon efficient cell penetration, reduced periplasmic ß-lactamase levels and, hence, the MIC. The CFX–DOTAU NP did not modify the MIC either, indicating that the nanoformulation of CFX as an antibiotic reservoir, such as that which was previously reported in [25], was insufficient to enhance the antibiotic effect, and that ONα functionalization was required to decrease the CFX MIC.

The achieved MIC of 341 mg·L^−1^ of CFX corresponded to 50 µM Onα, which was of comparable range with the results described in the literature for Gram negative resistant bacteria, while keeping in mind that the sequences used, genes targeted and antibiotics co-administered were not the same. The DNA tetrahedron carrier of *bla*_CTX-M-3_ decreased the in vitro growth of cefotaxime-resistant *E. coli* at 40 µM in the presence of cefotaxime [38]. Negatively charged liposomes encapsulating PTO oligonucleotides targeting *oprM* (the outer membrane protein component of the efflux pump) restored the sensitivity to piperacillin of resistant *Pseudomonas aeruginosa* at 2–20 µM concentration [68]. The same concentration of PTO sequences targeting *acrB* encapsulated in negatively charged liposomes significantly decreased the MIC value of ciprofloxacin in fluoroquinolone-resistant *E. coli* [69].

## 4. Conclusions

Our results highlighted the potential of the nucleolipid carrier DOTAU to deliver, in the same formulation, the antibiotic, CFX, along with ONα sequences to decrease the bacterial resistance of ESBL-producing *E. coli*, a WHO priority pathogen. ONα NP with the molar ratio of 10:24:1 were able to efficiently decrease the CFX MIC by 75%. This nanoformulation strategy can be considered as a relevant and efficient strategy for oligonucleotide intra-bacterial delivery to fight against antibiotic resistances. The future extensions of this work include testing the formulation of different resistant bacterial *E. coli* strains and other bacterial species producing CTX-M-15, along with further pharmaceutical development.

## Figures and Tables

**Table 1:** Yield of ion pair formation at different ratios of CFX–DOTAU during formulation.

Weight Ratio CFX–DOTAU	Molar Ratio CFX–DOTAU	Ion Pair Yield (%)
1:1	1.3:1	68.9 ± 5.0
1:1.3	1:1	80.4 ± 3.7
1.9:1	2.3:1	32.6 ± 3.2

**Table 2:** IR vibrational bands of CFX, DOTAU and CFX–DOTAU ion pair.

Chemical Group Considered	DOTAU (cm^−1^)	CFX (cm^−1^)	Ion Pair (cm^−1^)
C=N—OCH_3_		1533	1536
C=N—OCH_3_		1533	1536
C=N		1498	1500
C=N		1602	1602
N-(C=O)-N	1690		1690
CH2	1462		1464
CH3	1380		1382

**Table 3:** Nanoprecipitation protocol optimization in view of colloidal properties and encapsulation yield of CFX–DOTAU NP.

V Methanol (µL)	V Water (mL)	Size ± SD (nm)	ZP ± SD (mV)	PDI ± SD	NP Yield (%)
300	10	150.1 ± 1.0	44.5 ± 6.5	0.141 ± 0.010	64
500	10	123.3 ± 0.7	39.4 ± 3.9	0.169 ± 0.004	64
300	5	137.4 ± 2.3	34.7 ± 1.3	0.127 ± 0.010	57

**Table 4:** Physico-chemical properties of CFX–DOTAU NPs and ONα NPs.

Formulation	Mean Size ± SD (nm)	Mean PDI ± SD	Mean Zeta ± SD (mV)
CFX–DOTAU NP	157.6 ± 7.6	0.160 ± 0.050	35.9 ± 3.0
ONα NP	187.1 ± 21.1	0.191 ± 0.065	−45.8 ± 11.2

## Data Availability

The data are available from the corresponding author upon request.

## Supplementary Materials

The following are available online at https://www.mdpi.com/1999-4923/14/2/299/s1, Figure S1: Infrared spectra of DOTAU, CFX and CFX–DOTAU ion pair, Figure S2: TEM images and zoom on a single particle of (a) ONα_600_ NP and (b) CFX–DOTAU NP; (c) STEM compositional mapping of ONα_600_ NP and (d) intensity counted by energy dispersive-X-ray spectroscopy for bands specific of C, N, O, P, S atoms for ONα_600_ NP (zone 1 on HAADF image), CFX–DOTAU NP and Background (zone 2 on HAADF image), Table S1: NP size, PDI and zeta potential (mean ± SD, n = 6) upon sample dilution for CFX–DOTAU and ONα NP.
