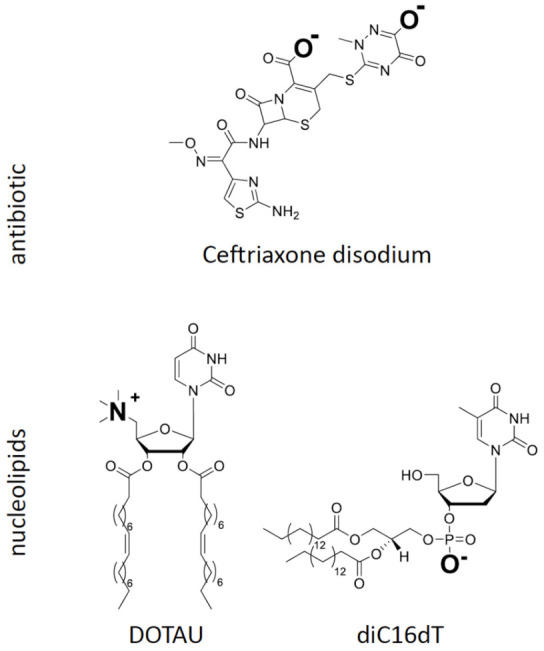# Correction: Nguyen et al. Oligonucleotide Solid Nucleolipid Nanoparticles against Antibiotic Resistance of ESBL-Producing Bacteria. *Pharmaceutics* 2022, *14*, 299

**DOI:** 10.3390/pharmaceutics14112317

**Published:** 2022-10-28

**Authors:** Phuoc Vinh Nguyen, Clémentine Aubry, Narimane Boudaoud, Alexandra Gaubert, Marie-Hélène Langlois, Mathieu Marchivie, Karen Gaudin, Corinne Arpin, Philippe Barthélémy, Tina Kauss

**Affiliations:** 1 ARNA, Inserm U1212, CNRS 5320, University of Bordeaux, 146 Rue Léo Saignat, CEDEX, 33076 Bordeaux, France; 2 UMR 5026, University of Bordeaux, CNRS, Bordeaux-INP, ICMCB, 87 Avenue du Dr Albert Schweitzer, CEDEX, 33608 Pessac, France; 3 MFP, CNRS 5234, University of Bordeaux, 146 Rue Léo Saignat, CEDEX, 33076 Bordeaux, France

## Error in Figure

In the original publication [1], there was a mistake in **Figure 1** as published. **The chemical formulae of nucleolipids, i.e., DOTAU and diC16dT, did not display correctly, as the repeats of carbon atoms in the lipid chain (parenthesis and number of repeats) were missing.** The corrected **Figure 1** appears below. The authors apologize for any inconvenience caused and state that the scientific conclusions are unaffected. This correction was approved by the Academic Editor. The original publication has also been updated.