# Supplementary material for: Oligonucleotide Solid Nucleolipid Nanoparticles against Antibiotic Resistance of ESBL-Producing Bacteria
Source: Pharmaceutics. 2022 Jan 27;14(2):299. doi: 10.3390/pharmaceutics14020299 (PMC8876242; doi:10.3390/pharmaceutics14020299)
Supplement: Supplementary file 1 [file pharmaceutics-14-00299-s001.zip › pharmaceutics-1523529-supplementary.pdf]

pharmaceutics

MDPI

Supplementary information of the article

Oligonucleotide solid nucleolipid nanoparticles against antibiotic resistance of ESBL-producing bacteria

Phuoc Vinh Nguyen, Clémentine Aubry, Narimane Boudaoud, Alexandra Gaubert, Marie-Hélène Langlois, Mathieu Marchivie, Karen Gaudin, Corinne Arpin, Philippe Barthélémy and Tina Kauss

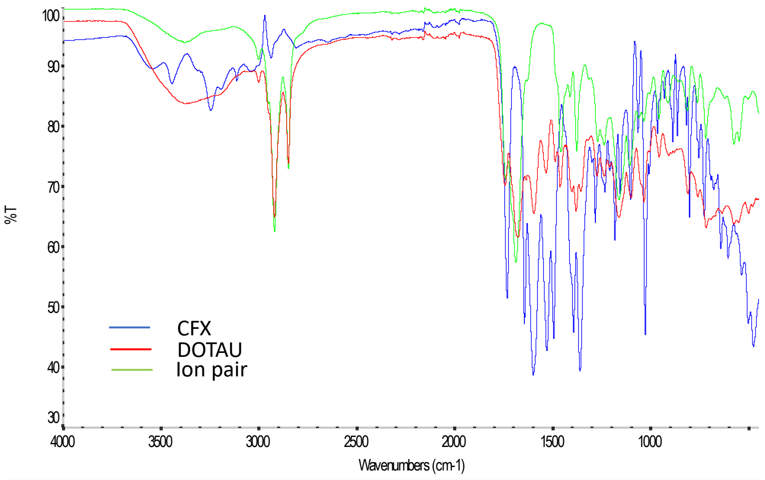

Figure S1. Infrared spectra of DOTAU, CFX and CFX–DOTAU ion pair.

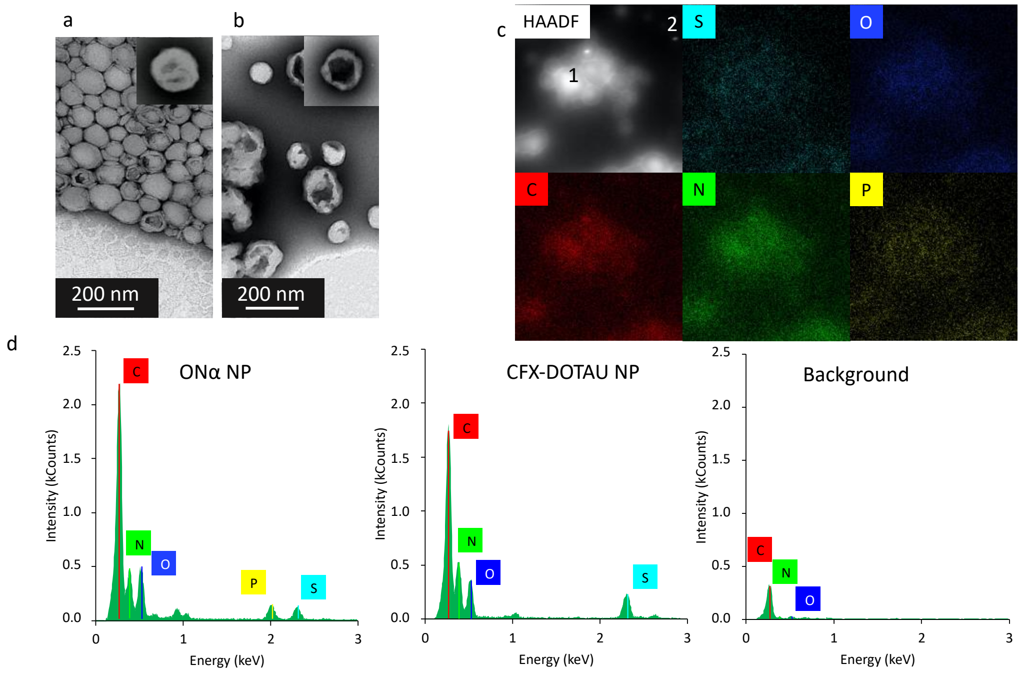

Figure S2. TEM images and zoom on a single particle of (a) ONα$_{600}$ NP and (b) CFX–DOTAU NP; (c) STEM compositional mapping of ONα$_{600}$ NP and (d) intensity counted by energy dispersive-X-ray spectroscopy for bands specific of C, N, O, P, S atoms for ONα$_{600}$ NP (zone 1 on HAADF image), CFX–DOTAU NP and Background (zone 2 on HAADF image).

Table S1. NP size, PDI and zeta potential (mean ± SD, n=6) upon sample dilution for CFX–DOTAU and ONα NP.

NP Formulation	Dilution	Size (nm)	PDI	Zeta (mV)
CFX–DOTAU NP	1/100	155.9 ± 1.6	0.137 ± 0.016	35.9 ± 1.1
	1/200	157.1 ± 2.1	0.146 ± 0.016	39.2 ± 2.6
	1/500	160.0 ± 2.3	0.181 ± 0.024	31.2 ± 8.1
	1/1000	167.4 ± 11.0	0.208 ± 0.054	31.2 ± 11.9
ONα NP	1/100	185.2 ± 8.7	0.158 ± 0.036	-55.2 ± 0.9
	1/200	182.1 ± 13.3	0.157 ± 0.049	-53.4 ± 4.8
	1/500	183.2 ± 14.7	0.157 ± 0.055	-48.9 ± 5.7
	1/1000	180.0 ± 7.2	0.164 ± 0.029	-45.4 ± 4.6
